# The complete mitochondrial genome of the pentastomid *Linguatula arctica* (Pentastomida) from reindeer (*Rangifer tarandus*) in Northern Norway

**DOI:** 10.1080/23802359.2020.1823255

**Published:** 2020-10-15

**Authors:** José Horacio Grau, Jason A. Dunlop, Martin Meixner, Dennis Tappe, Bjørn Gjerde

**Affiliations:** a Leibniz-Institut für Evolutions- und Biodiversitätsforschung, Museum für Naturkunde Berlin, Berlin, Germany; b SMB Services in Molecular Biology GmbH, Ruedersdorf, Germany; c Bernhard-Nocht-Institut für Tropenmedizin, Nationales Referenzzentrum für tropische Infektionserreger, Hamburg, Germany; d Faculty of Veterinary Medicine, Department of Food Safety and Infection Biology, Norwegian University of Life Sciences, Sentrum, Oslo, Norway

**Keywords:** *Linguatula arctica*, tongue worm pentastomid parasite

## Abstract

Here, we present the first complete mitochondrial genome of the pentastomid *Linguatula arctica* collected from the nasal passages of a reindeer (*Rangifer tarandus*) in Norway. The full length mitochondrial genome of *L. arctica*, which measures 14,789 bp in length, contains 13 protein-coding genes, 2 ribosomal RNA genes and 22 transfer RNA genes. A clear A + T bias is observed in the mitogenome of *L. arctica* with an overall base composition of 32.6% A, 27.5% T, 32.8% C, and 7,1% G., and a GC content of 39.9%. The gene arrangement is identical to that of previously described pentastomid mitogenomes.

Pentastomida are an intriguing group of highly adapted worm-like hematophagous parasitic crustaceans (Riley 1986). The pentastomid genus *Linguatula* usually infests the nasal sinuses of carnivorous mammals belonging to the families Canidae, Hyaenidae and Felidae while using other mammals, particularly ruminants (Rezaei et al. 2011), as intermediate hosts. In fact, a wide range of mammals have been recorded as intermediates (Christoffersen & De Assis 2013) and humans can also occasionally be affected. The boreal species, *L. arctica*, parasitizes the upper respiratory (nasal) passages and maxillary sinuses of reindeer and caribou and is sometimes referred informally to as the ‘reindeer sinus worm’. For an overview of its morphology and biology, see Nikander and Saari (2006) and Riley et al. (1987). An interesting aspect is thus the fact that, unlike the other four *Linguatula* species, it appears to have a direct life cycle (Haugerud and Nilssen 1990). In other words, *L. arctica* does not require an intermediate host and, unusually, has an ungulate – as opposed to a carnivore – as its definitive host.

DNA for the present study was obtained from an adult female *L. arctica* specimen that had been collected from the nasal passages of a semi-domesticated reindeer in the municipality of Tromsø (69°39′50″N, 18°58′02″E), northern Norway (Gjerde 2013). The complete mitogenome was obtained from next-generation shotgun sequencing. Paired-end Illumina sequencing libraries were generated from tissue sample and sequenced on an Illumina NextSeq 500 platform, using Illumina NextSeq® 500/550 High Output Kit V2. Sequencing yielded over 2 million 150 bp paired end reads. A complete mitochondrial genome was obtained with NOVOplasty 2.4 (Dierckxsens et al. 2017) using kmer 51, and the mitogenome of *Linguatula serrata* (MG951756) as bait reference.

Annotations were carried out with MITOchondrial genome annotation Server (MITOS) (Bernt et al. 2013), and manual validation of the coding regions using the NCBI ORF Finder (http://www.ncbi.nlm.nih.gov/gorf/gorf.html) in combination with NCBI’s Conserved Domain Database (CDD) (Marchler-Bauer et al. 2017). The annotated sequence file was submitted to NCBI (accession MN792849), and a voucher of the specimen is depostied in the Museum für Naturkunde Berlin under the accession code ZMB_Arach 49469. The phylogenetic position of the new sequence of *L. arctica* according to the gene Cytochrome B is shown in Figure 1.

**Figure 1.**
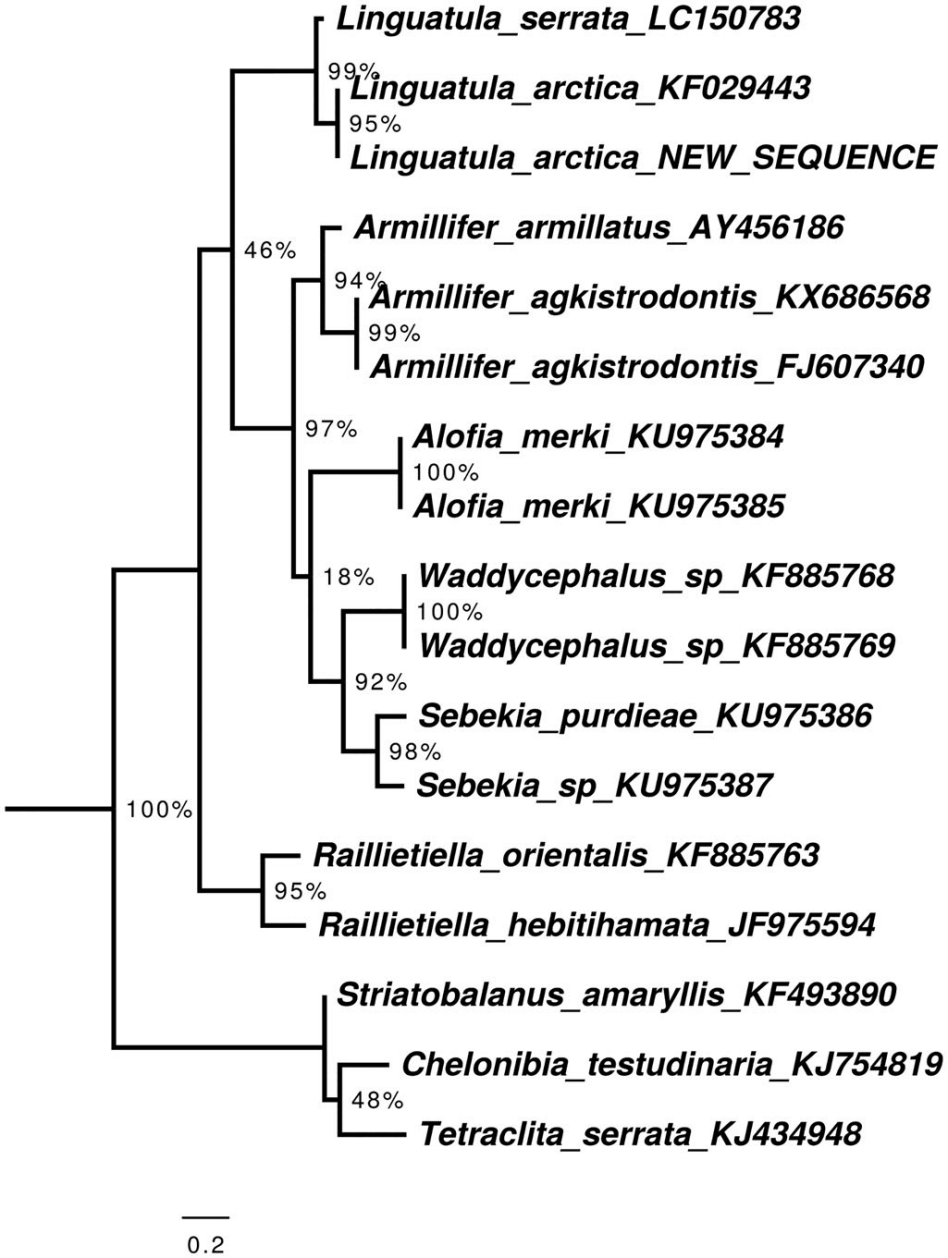
Maximum likelihood tree illustrating the phylogenetic position of the newly sequenced Linguatula arctica gene sequence among a subset of pentastomid species. Cytochrome oxidase I sequences were aligned using MAFFT 7.271 and highly divergent or poorly aligned regions were removed with GBlocks 0.91 b (Castresana 2000) allowing for gap positions and smaller blocks. Trees were calculated using PhyML 3.1 (Guindon et al. 2010) with 12 rate categories, optimized equilibrium frequencies, GTR model of sequence evolution and combined heuristics (Nearest Neighbor Interchange and Subtree Pruning and Rerafting). Branch support was calculated using approximate likelihood ratio tests as implemented in PhyML.

The complete mitochondrial transcript of *L. arctica* was 14,789 bp in length and contained 13 protein-coding genes (PCGs), 2 ribosomal RNA genes and 22 transfer RNA genes. As described for other related pentastomid mitogenomes (Grau et al. 2017; Naude et al. 2018), the mitochondrial genome of *L. arctica* contained an A + T bias with an overall base composition of 32.6% A, 27.5% T, 32.8% C, and 7.1% G. The gene arrangement of the present mitogenome is identical to those of other pentastomids (Lavrov et al. 2004; Li et al. 2016; Grau et al. 2017; Naude et al. 2018).

Most of the genes are encoded on the L-strand with the exception of four protein-coding genes (*NAD5*, *NAD4*, *NAD4L*, *NAD1*), nine tRNA (*tRNA^Cys^*, *tRNA^Gln^*, *tRNA^Tyr^*, *tRNA^Phe^*, *tRNA^His^*, *tRNA^Thr^*, *tRNA^Pro^*, *tRNA^Val^*, *tRNA^LeuCUN^*) and both rRNAs (12S and 16S), which were encoded in the H-strand.

Seven PCGs (*ND2*, *COX1*, *ATP8*, *ATP6*, *ND3, ND5* and *ND4L*) had ATA as initiation codon, while four PCGs (*COX3*, *ND4*, *CYTB* and *ND1*) presented ATG as initiation codon. An alternative initiation codon ATT was found for *COX2* and *ND6*. Incomplete termination codons were found for three PCGs (*COX2*, *COX3* and *ND5*) which are complemented with additional 3′-A residues. Nine PCGs (*ND2, COX1, ATP8*, *ATP6*, *ND3*, *ND4*, *ND6, CYTB and ND1*) used a TAA termination codon, while *ND4L* used a TAG termination codon. The 12S and 16S genes had a length of 655 and 1,126 bp, respectively.

The mitochondrial genome of *L. arctica* contains a very small control region in comparison with other pentastomid mitogenomes, and confirmation of circularity and absolute length of the control region are difficult to establish precisely. Nonetheless, we expect this mitogenome will aid the accurate classification of this species – traditionally misidentified as *L. serrata* (see comments in Nikander and Saari 2006) – as well as parasite diagnostics in general.

## Data Availability

The data that support the findings of this study are openly available in nucleotide database of NCBI (National Center for Biotechnology Information) at https://www.ncbi.nlm.nih.gov, accession number MN792849.
